# Mediating Effect of IADL and Depression in the Relationship Between Ability to Perform Movements and Death Preparation in Elderly with Osteoarthritis or Rheumatoid Arthritis

**DOI:** 10.3390/healthcare13050513

**Published:** 2025-02-27

**Authors:** Myoungjin Kwon, Sun Ae Kim, Jiyoung Kim

**Affiliations:** 1 Department of Nursing, Daejeon University, Daejeon 34520, Republic of Korea; mjkwon@dju.kr; 2 Department of Nursing, Korea National University of Transportation, Jeungpyeong-gun 27909, Republic of Korea; 3 Department of Nursing, Woosuk University, Wanju-gun 55338, Republic of Korea

**Keywords:** death preparation, depression, ability to perform movements, instrumental activities of daily living, osteoarthritis, rheumatoid arthritis

## Abstract

**Background/Objectives**: This study investigated the mediating effects of instrumental activities of daily living (IADL) and depression on the relationship between the ability to perform movements and death preparation among older adults with osteoarthritis (OA) or rheumatoid arthritis (RA). **Methods**: This study is a secondary analysis of data from the Ministry of Health and Welfare of Korea’s 5th (2020) survey of the elderly. It included 1483 elderly individuals with OA or RA. We measured the ability to perform movements, death preparation, IADL, and depression using validated scales. Hayes’ PROCESS Macro for SPSS model 6 was used to determine the mediating effects. **Results**: Death preparation exhibited significant differences based on satisfaction with economic conditions, fall experiences, and suicidal ideation. A significant correlation existed between the ability to perform movements, death preparation, IADL, and depression. IADL demonstrated a significant mediating effect between the ability to perform movements and death preparation. However, depression did not exhibit a significant mediating effect. **Conclusions**: IADL significantly mediates the relationship between movement ability and death preparation in elderly individuals with OA or RA. Lower movement ability was associated with higher death preparation and greater IADL dependence. However, contrary to expectations, depression did not show a significant mediating effect. These findings offer significant insights for developing interventions to enhance the quality of life and prepare for dignified death in elderly individuals with OA or RA. Future longitudinal studies are required to clarify the causal relationships and evaluate the efficacy of IADL enhancement programs.

## 1. Introduction

### 1.1. Background

Death preparation is a critical process in which individuals accept the final stage of life and maintain their quality of life through emotional and practical readiness. Chronic diseases are a leading cause of mortality, making death preparation for individuals with chronic conditions an essential issue [1]. In particular, the deterioration of Activities of Daily Living (ADL) in older adults with chronic diseases can serve as a significant factor necessitating more immediate and deliberate consideration of death preparation [2].

Arthritis (OA) is a prevalent chronic musculoskeletal disorder affecting the elderly population worldwide [3]. Osteoarthritis (OA), primarily affecting the knee and hip joints, is a leading cause of disability in the elderly, impacting approximately 20% of adults aged 60 and older [4]. Similarly, Rheumatoid Arthritis (RA), an autoimmune inflammatory disease with a global prevalence of 0.5% to 1.3%, predominantly targets the joints and often leads to severe disability [5]. Arthritis, a common chronic musculoskeletal disorder among older adults, often results in significant physical limitations due to chronic pain, stiffness, and functional impairments, which can severely impact their ability to perform daily activities and maintain independence [6]. These limitations extend beyond physical discomfort, severely impairing Instrumental Activities of Daily Living (IADL) and negatively impacting psychological health by increasing the risk of depression [7]. Moreover, depression is closely associated with pain, which is one of the most common symptoms of arthritis [8]. Such challenges are likely to disrupt an individual’s ability to engage in death preparation—a critical process for maintaining quality of life at the end stage of life [9]—by exacerbating feelings of helplessness and social isolation. Given these impacts, it is essential to clarify the mediating roles of IADL and depression in the relationship between mobility and death preparation among arthritis patients.

A previous study [2] found that limitations in ADL or IADL did not directly predict levels of preparation for death, suggesting that death preparation is more strongly influenced by psychological and social factors rather than physical functional limitations. Although limitations in IADL may not directly predict levels of death preparation, they can exert an indirect influence through their impact on mental health. Impairments in IADL often lead to increased risks of depression and social isolation, as the loss of functional independence can foster feelings of helplessness and disconnection from others. These psychological challenges, in turn, may hinder individuals from engaging in meaningful death preparation activities, such as future care planning or communicating end-of-life preferences. Therefore, it is crucial to explore the mediating roles of psychological factors, such as depression and social isolation, in the relationship between mobility limitations and death preparation, particularly among older adults with chronic conditions like arthritis.

Depression is a significant psychological factor that can profoundly influence death-related decision-making and preparation in patients with chronic illnesses, including arthritis. Patients with depression often experience diminished emotional resilience, impaired cognitive function, and reduced capacity to engage in proactive planning for end-of-life care. These challenges may hinder their ability to articulate preferences regarding medical interventions, resolve personal or familial concerns, or make informed decisions about future care [10]. In arthritis patients, the interplay between chronic pain and depression is particularly pronounced. Studies have shown that knee osteoarthritis (OA) patients have a 10.4% prevalence of moderate to severe depression, reflecting the substantial mental health burden associated with this condition. Additionally, rheumatoid arthritis (RA) and OA have been identified as significant risk factors for major depressive disorder (MDD), likely due to the combined effects of persistent pain, functional limitations, and reduced quality of life [11]. Given that depression exacerbates physical symptoms and diminishes overall well-being, it may further impair patients’ ability to engage in meaningful discussions about death-related preparations [10].

However, rather than being solely a source of distress, the contemplation of mortality triggered by chronic pain and depression in arthritis patients may provide an opportunity to engage in meaningful death preparation, which can help address these psychological challenges. This opportunity for meaningful death preparation offers significant benefits, including alleviating death-related anxiety and stress, fostering a constructive awareness of death, and motivating individuals to value their lives [12]. These advantages are significant for patients with arthritis who frequently experience chronic pain and depression. Physical discomfort and emotional distress due to arthritis may lead patients to reflect more intensely on their mortality than the general population. Although research specifically addressing death preparation among patients with arthritis is very limited and almost impossible to find, it is evident that the mental health challenges associated with arthritis, particularly depression, can prompt individuals to contemplate death. This contemplation may naturally evolve into considerations regarding how to prepare for the end of life, making death preparation an essential component of holistic care for these patients.

The ability to perform movements is a fundamental determinant of physical independence and autonomy in older adults, influencing their capacity to engage in meaningful death preparation. However, the mechanisms underlying this relationship remain unclear, particularly in the context of arthritis, where physical limitations and psychological challenges are deeply intertwined. By examining the mediating roles of IADL and depression, this study aims to bridge this gap in understanding and provide a comprehensive framework for addressing the unique needs of elderly individuals with osteoarthritis or rheumatoid arthritis. This approach will not only deepen our understanding of the interplay between mobility, functional independence, and psychological health but also inform targeted interventions to enhance death preparation and overall quality of life in this vulnerable population.

### 1.2. Purpose of This Study

Previous studies have primarily focused on the individual effects of IADL or depression on mobility and quality of life in older adults. However, few have examined the specific dynamics between movement ability, death preparation, and the mediating roles of IADL and depression, particularly in the context of arthritis.

This study aims to investigate the mediating effects of instrumental activities of daily living (IADL) and depression on the relationship between the ability to perform movements and death preparation in elderly individuals with osteoarthritis (OA) or rheumatoid arthritis (RA). While previous research has examined the individual effects of physical and psychological factors on death preparation in older adults, limited attention has been given to how these variables interact in the context of chronic musculoskeletal conditions. By employing a mediation model, this study seeks to clarify the pathways through which IADL and depression influence the relationship between movement ability and death preparation. Specifically, it explores how functional independence (IADL) and psychological well-being (depression) act as mediators in shaping the extent to which physical limitations impact death preparation. This approach provides a comprehensive framework for understanding the complex interplay between physical health, functional capacity, and psychological outcomes in end-of-life readiness.

Ultimately, this study aims to contribute to the development of tailored interventions that address both functional and psychological needs, enhancing death preparation for elderly individuals with OA or RA.

## 2. Materials and Methods

### 2.1. Study Design

This study is a descriptive research study that analyzed data obtained from the 5th (2020) Elderly Survey conducted by the Ministry of Health and Welfare. This descriptive study aimed to identify the mediating effects of IADL and depression on the relationship between the ability to perform movements and death preparation in elderly patients with OA or RA.

### 2.2. Participants

This study utilized data from the 5th (2020) survey of the elderly. The survey on the elderly is a statutory survey in accordance with Article 5 of the Elderly Welfare Act and has been administered triennially since its enactment in 2007. It offers fundamental data for determining policy measures that promote a healthy and active life in old age by identifying the actual conditions of old age, such as health, economy, social participation, and leisure. In 2020, according to the designed sampling method, a survey was conducted targeting older adults aged 65 years and above in 969 survey districts. The number of people who completed the final survey was 10,097. The participants of this study were 1483 elderly individuals with OA or RA.

### 2.3. Study Variables

#### 2.3.1. Ability to Perform Movements

The ability to perform movements comprised six tasks: running one lap (400 m) around the playground; walking one lap (400 m) around the playground; climbing 10 steps without pausing, bending, squatting, or kneeling; reaching for an object above head height; and lifting or relocating an object weighing approximately 8 kg [13]. The scores range from 6 to 24 on a 4-point Likert scale. The higher the score, the worse the activity required to perform the movement [14].

#### 2.3.2. Death Preparation

Death preparation encompassed eight items: participation in death preparation education; discussions about inheritance with family members; funeral intentions; funeral counseling and membership in mutual aid associations; writing a will; writing prior statements on life-sustaining treatment, pledges for organ donation, preparation of burial grounds (including delayed burial grounds, sealing halls, and graveyards); and preparing veterinarians or portrait photos. This scale is scored on a dichotomous scale, with 1 point assigned for preparedness and 0 points for lack of preparation. A higher score indicates greater preparation for death.

#### 2.3.3. IADL

The IADL was measured using the K-IADL (Korean-instrumental activities of daily living) tool developed by Won et al. [15]. With 10 questions, dressing, grooming, housework, meal preparation, laundry, taking medicine, and money management were measured on a 3-point Likert scale ranging from 1 (complete independence) to 3 (complete assistance), while shopping, making and receiving phone calls, and using transportation were measured on a 4-point Likert scale ranging from 1 (complete independence) to 4 (complete assistance). A higher score indicates greater dependence on daily life performance ability.

#### 2.3.4. Depression

Depression was measured using the Korean Short Form of the Geriatric Depression Scale (SGDS-K), which was developed by Yesavage et al. [16] and standardized by Kee [17]. The scale is a dichotomous one with 15 questions; each question requires a “yes” or “no” response. One point is assigned for “yes” and 0 points for “no”. Five items with reversed content were reverse coded, and the total score ranged from 0 to 15, with higher scores indicating greater severity of depression.

### 2.4. Ethical Considerations

The 2020 Elderly Survey was conducted through one-on-one interviews between 14 September and 20 November 2020. The 2020 Elderly Survey obtained approval from the National Statistical Office for statistical modifications to the sample design and survey content (approval number 117071). The investigation was conducted with approval from the Korea Institute for Health and Social Affairs Bioethics Committee (IRB) (Korea Institute for Health and Social Affairs Bioethics Committee (IRB) review result notice (No. 2020-36)).

### 2.5. Analysis

The study data were analyzed using SPSS/WIN v.25.0, with a significance threshold established at 0.05. Descriptive statistics were employed to analyze general characteristics and the degree of major variables, while t-tests and ANOVA were utilized to assess differences in death preparation based on general characteristics. Scheffé’s test was performed for post hoc analysis. Correlations between the ability to perform movements, death preparation, IADL, and depression were analyzed using Pearson’s correlation coefficients. Hayes’ [18] PROCESS Macro for SPSS v.3.5 model 6 was utilized to determine the mediating effect of IADL and depression on the relationship between the ability to perform movements and death preparation. The significance of the mediating effect was determined by bootstrapping (10,000) to verify the indirect effect.

## 3. Results

### 3.1. General Characteristics Based on Death Preparation

There was a significant difference in death preparation according to satisfaction with economic conditions, fall experience in the past year, and suicidal ideation. The more economically satisfied the subjects were, the better they prepared for death, and subjects who had a fall experience and suicidal ideation prepared for death better (Table 1).

### 3.2. Correlation Between the Ability to Perform Movements, Death Preparation, IADL, and Depression

As the ability to perform movements score increased, the death preparation, IADL, and depression scores also increased. That is, as the ability to perform activities worsens, preparation for death improves and IADL and depression worsen. Further, the better the death preparation, the worse the IADL and depression, and the worse the IADL, the worse the depression (Table 2).

### 3.3. Mediating Effect of IADL and Depression on the Relationship Between the Ability to Perform Movements and Death Preparation

The mediating effect of IADL and depression on the relationship between the ability to perform movements and death preparation was analyzed using Model 6 of the PROCESS Macro for SPSS v.4.0.

**Figure 1:**
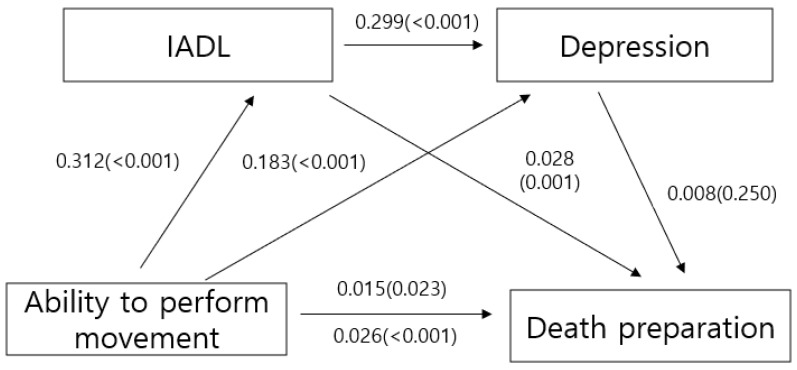
Mediating effect of variables.

The results show that each model was statistically significant (see Table 3 and Figure 1). In Stage 1, the ability to perform movements significantly influenced IADL (B = −0.312, *p* < 0.001). In Stage 2, the ability to perform movements (B = 0.183, *p* < 0.001) and IADL (B = 0.299, *p* < 0.001) significantly affected depression. In Stage 3, the ability to perform movements (B = −0.015, *p* = 0.023) and IADL (B = 0.028, *p* = 0.001) significantly affected death preparation. In Stage 4, the ability to perform movements significantly affected death preparation (B = 0.026, *p* < 0.001). The overall effect between the ability to perform movements and death preparation decreased from 0.026 (*p* < 0.001) to 0.015 (*p* = 0.023) when two parameters, IADL and depression, were included (Table 4). Therefore, it was established that IADL and depression exerted negative effects on death preparation; that is, an indirect mediating impact.

To determine the significance and influence of the double mediation effect, the distinctions among the three pathways—namely, the path with only the first parameter input, the path with only the second parameter, and the effect of the path with both the first and second parameter inputs—were analyzed. To verify the significance of the mediating effect, a bootstrapping method was employed, and 10,000 samples were resampled. Statistical significance was determined utilizing 95% confidence intervals (CI).

Table 5 shows that the route from the ability to perform movements to death preparation through IADL demonstrated a significant mediating effect (B = 0.009, 95% CI: 0.002–0.163). The path from the ability to perform movements to death preparation via depression (B = 0.001, 95% CI: −0.001–0.004) and the ability to perform movements to death preparation via IADL and depression exhibited no significant mediating effect (B = 0.001, 95% CI: −0.001–0.002). Therefore, IADL was a significant parameter in the relationship between the ability to perform movements and death preparation, although depression was not a significant parameter.

## 4. Discussion

This study aimed to validate the mediating effect between IADL and death preparation in older adults with OA or RA, utilizing data from the 5th survey on the elderly (2020) conducted by the Ministry of Health and Welfare of the Republic of Korea.

The results of this study revealed significant differences in death preparation among older adults with OA or RA based on their satisfaction with economic conditions, fall experiences, and suicidal ideation. Hwang et al. (2022) found that economic status significantly influenced the intention to receive life-sustaining treatment among the elderly; the lower the satisfaction with economic conditions, the higher the life-sustaining treatment intention [19]. Fall experience is a significant factor affecting daily living ability, and it may indirectly impact death preparation by elevating the risk of depression [20,21,22]. The prevalence of suicidal ideation in patients with OA exceeds that of the general population, and the presence of physical multimorbidity increases the risk of suicidal ideation and suicide attempts [23].

The results indicate that the mediating effect of IADL was significant in the relationship between the ability to perform movements and death preparation in older patients with OA or RA. This indicates that the ability to perform movements correlates with a higher dependence on IADL and an increase in death preparation. In elderly patients with OA or RA, a decline in physical function is significantly associated with an increase in IADL dependence. In elderly patients with OA or RA, the risk of IADL disability is higher than in the general elderly population [24], and this decline in physical function is significantly associated with increased IADL dependence. This dependence reduces the autonomy of older adults and increases their awareness of their own limitations, which triggers consideration of death and death preparation [24,25,26]. Additionally, the decline in physical capacity may make older adults vulnerable, which may lead to psychological changes in death preparation [27,28,29]. Thus, declining physical function, especially the decreased ability to perform movements, correlates with increased death preparation in older adults [30], indicating that as physical limitations increase, older adults tend to recognize and prepare for their own mortality better. Older adults with chronic diseases, such as OA or RA, are more inclined to contemplate death preparation compared to the general elderly population [31]. IADL plays a pivotal role as a mediator between movement ability and death preparation, as it reflects the functional independence of older adults. This highlights that IADL may exert a more direct influence on practical aspects of death preparation by fostering autonomy and self-reliance.

In contrast, depression primarily reflects psychological conditions, which may have a less pronounced impact on specific behavioral outcomes like death preparation. Depression was not a major mediator in the relationship between the ability to perform movements and death preparation in older adults with OA or RA. Previous studies indicated a significant correlation between depression and frailty in community-dwelling older adults [32]; however, this association was not specific to OA or RA. A systematic literature review on OA and depression reported that while depression can affect functional outcomes in osteoarthritis (OA) patients, its influence is highly context-dependent and varies across different scenarios [33]. Additionally, it has been reported that depression may contribute to heightened death anxiety but does not necessarily translate into active death preparation behaviors [34]. The finding that the mediating effect of depression between the ability to perform movements and death preparation was not significant implies that the effect of depression may be limited in this specific relationship. This is because factors such as IADL may have a more significant mediating influence because of the nature of the death preparation variable. Many studies on depression and aging focus on community-dwelling older adults or clinical populations without adequately distinguishing between those with OA/RA and the general elderly population [24]. In addition, due to the nature of the study, it should be considered that bias may occur due to the inability to control confounding variables such as social support or chronic pain levels [34,35]. Therefore, additional research is required to clarify the relationship between depression, ability to perform movements, and death preparation in patients with OA or RA. Moreover, it is necessary to analyze the role of depression in the process of concretizing death preparation behaviors further. Additionally, future research should consider other influencing variables to provide a more comprehensive understanding. To address these limitations comprehensively, we recommend that future research integrates both quantitative and qualitative methodologies. Moreover, qualitative interviews could provide deeper insights into individual experiences of death preparedness and complement quantitative findings from structured datasets. Additionally, longitudinal primary data collection could offer more flexibility in exploring causal relationships between mobility, IADL, depression, and death preparedness.

This study indicated the mediating role of IADL in the relationship between the ability to perform movements and death preparation in older adults with OA and RA. This indicates that maintaining independent physical function in the elderly is important in preparing for death. Additionally, satisfaction with economic conditions, fall experiences, and suicidal ideation were significantly related to death preparation, indicating that both physical health and economic and psychological factors are crucial in death preparation. Contrary to expectations, the absence of a significant mediating effect of depression indicates that functional independence is more critical than depression in the death preparedness of older adults with arthritis.

This study has several limitations that should be addressed in future research. First, because this study was designed and conducted as a cross-sectional study using secondary data analysis, future research could consider longitudinal studies to elucidate causal relationships among key variables, as well as mixed methods approaches integrating both quantitative and qualitative research methodologies. Additionally, this study did not distinguish between OA and RA characteristics and focused on older Korean adults, which may limit generalizability. Moreover, the results of this study indicate the need to develop and verify a more comprehensive model that includes other potential mediating factors affecting death preparation.

Lastly, the absence of data on spiritual dimensions, such as religious practices and existential life review, and the exclusion of family or community-level variables, restrict the comprehensive understanding of death preparation. Consequently, future research may consider integrating spiritual dimensions into conceptualizing end-of-life preparedness to provide a more holistic perspective.

## 5. Conclusions

This study indicated that IADL serves a significant mediating role in the correlation between the ability to perform movements and death preparation in elderly patients with OA or RA. The main results indicated that a lower ability to perform movements correlated with a higher level of death preparation, higher IADL dependence, and more severe depression. IADL demonstrated a significant mediating effect between the ability to perform movements and death preparation. Contrary to expectations, depression did not exhibit a significant mediating effect.

This study offers critical data for establishing policies aimed at enhancing the quality of life and preparing for a dignified death for elderly patients with OA or RA. Future studies should clarify the causality of this relationship through longitudinal studies and verify the effectiveness of IADL enhancement programs.

## Figures and Tables

**Table 1:** Differences in death preparation according to general characteristics (N = 1483).

Characteristics	Categories	N (%)	Death Preparation	
			M ± SD	t/F(*p*)
			Scheffé	
Gender	Male	244 (16.5)	1.15 (1.10)	1.02 (0.307)
	Female	1239 (83.5)	1.07 (1.01)	
Education level	≤Elementary school	930 (62.7)	1.09 (1.01)	0.22 (0.795)
	Middle school	290 (19.6)	1.05 (1.02)	
	≥High school	263 (17.7)	1.09 (1.07)	
Satisfaction with economic conditions	Satisfied	357 (24.6)	1.16 (1.07)	4.13 (0.016)
	Moderate	668 (46.0)	1.12 (0.99)	
	Unsatisfied	427 (29.4)	0.97 (1.01)	
Living with spouse	Yes	663 (44.7)	1.13 (1.11)	1.56 (0.118)
	No	820 (55.3)	1.05 (0.93)	
Relationship satisfaction with spouse	Satisfied	405 (61.8)	1.06 (1.08)	2.01 (0.136)
	Moderate	214 (32.7)	1.25 (0.08)	
	Unsatisfied	36 (5.5)	1.16 (0.84)	
Relationship satisfaction with children	Satisfied	954 (68.5)	1.12 (1.04)	0.64 (0.523)
	Moderate	337 (24.2)	1.08 (1.02)	
	Unsatisfied	101 (7.3)	1.01 (0.94)	
Satisfaction with relationships with friends and community	Satisfied	743 (50.2)	1.10 (1.01)	0.29 (0.741)
	Moderate	565 (38.1)	1.08 (0.94)	
	Unsatisfied	142 (9.6)	1.03 (1.30)	
Intention for life-sustaining treatment	Yes	156 (10.8)	1.08 (0.96)	0.12 (0.99)
	No	1296 (89.2)	1.08 (1.02)	
Number of chronic diseases	1	175 (11.8)	0.97 (0.98)	2.14 (0.118)
	2	404 (27.2)	1.04 (1.03)	
	≥3	904 (61.0)	1.13 (1.02)	
Fall experience in the past year	Yes	177 (11.9)	1.26 (1.22)	2.07 (0.039)
	No	1306 (88.1)	1.06 (0.99)	
Suicidal ideation	Yes	50 (3.4)	1.54 (1.59)	2.05 (0.045)
	No	1402 (96.6)	1.07 (0.99)	

IADL: Instrumental Ability of Daily Living.

**Table 2:** Correlation between the ability to perform movements, death preparation, IADL, depression.

Variables	Ability to Perform Movement	Death Preparation	IADL	Depression
	r(*p*)	r(*p*)	r(*p*)	r(*p*)
Ability to perform movement	1			
Death preparation	0.11 (<0.001)	1		
IADL	0.41 (<0.001)	0.13 (<0.001)	1	
Depression	0.30 (<0.001)	0.08 (0.001)	0.35 (<0.001)	1

IADL: Instrumental Ability of Daily Living.

**Table 3:** Mediating effect of IADL and depression on the relationship between ability to perform movements and death preparation in older adults.

No.	Variables			B (Coeffect)	SE	*p*	95% CI		R^2^
							LLCI	ULCI	
1	Ability to perform movements	→	IADL	0.312	0.020	<0.001	0.272	0.352	0.139
2	Ability to perform movements	→	Depression	0.183	0.023	<0.001	0.136	0.229	0.159
	IADL	→	Depression	0.299	0.028	<0.001	0.244	0.355	
3	Ability to perform movements	→	Death preparation	0.015	0.006	0.023	0.002	0.028	0.024
	IADL	→	Death preparation	0.028	0.008	0.001	0.013	0.044	
	Depression	→	Death preparation	0.008	0.007	0.250	−0.005	0.022	
4	Ability to perform movements	→	Death preparation	0.026	0.006	<0.001	0.014	0.038	0.012

ADL: Ability of Daily Living, CI = Confidence interval; LLCI = Lower limit confidence interval; ULCI = Upper limit confidence interval.

**Table 4:** Total effect, direct and indirect effect.

	Effect	SE	*p*	95% CI	
				LLCI	ULCI
Total effect	0.026	0.006	<0.001	0.014	0.038
Direct effect	0.015	0.006	0.023	0.002	0.028

CI = Confidence interval; LLCI = Lower limit confidence interval; ULCI = Upper limit confidence interval.

**Table 5:** Validation of mediating effect (bootstrapping).

Variables		Effect	Boot SE	95% CI	
				LLCI	ULCI
Indirect 1	Ability to perform movements → IADL→ Death preparation	0.009	0.003	0.002	0.163
Indirect 2	Ability to perform movements → Depression→ Death preparation	0.001	0.001	−0.001	0.004
Indirect 3	Ability to perform movements → IADL → Depression → Death preparation	0.001	0.001	−0.001	0.002

ADL: Ability of Daily Living, CI = Confidence interval; LLCI = Lower limit confidence interval; ULCI = Upper limit confidence interval.

## Data Availability

The data presented in this study are available in [Korean Statistics Promotion Institute] at [https://mdis.kostat.go.kr/index.do] (accessed on 18 April 2022).
